# A critical review of microbiome-derived metabolic functions and translational research in liver diseases

**DOI:** 10.3389/fcimb.2025.1488874

**Published:** 2025-02-24

**Authors:** Raja Ganesan, Durairaj Thirumurugan, Saranya Vinayagam, Dong Joon Kim, Ki Tae Suk, Mahalaxmi Iyer, Mukesh Kumar Yadav, Dibbanti HariKrishnaReddy, Jyoti Parkash, Arvinder Wander, Balachandar Vellingiri

**Affiliations:** ^1^ Department of Biotechnology, Faculty of Science and Humanities, SRM Institute of Science and Technology, Tamil Nadu, India; ^2^ Department of Bioscience, Saveetha School of Engineering, Saveetha Institute of Medical and Technical Sciences (SIMATS), Saveetha University, Chennai, India; ^3^ Institute for Liver and Digestive Disease, Hallym University, Chuncheon, Republic of Korea; ^4^ Department of Microbiology, School of Basic Science, Central University of Punjab, Bathinda, Punjab, India; ^5^ Advanced Pharmacology and Neuroscience Laboratory, Department of Pharmacology, School of Health Sciences, Central University of Punjab, Bathinda, Punjab, India; ^6^ Neurochemistry and Neuroendocrinology Lab, Department of Zoology, Central University of Punjab, Bathinda, Punjab, India; ^7^ Department of Pediatrics, All India Institute of Medical Sciences (AIIMS), Bathinda, Punjab, India; ^8^ Human Cytogenetics and Stem Cell Laboratory, Department of Zoology, School of Basic Sciences, Central University of Punjab, Bathinda, Punjab, India

**Keywords:** microbiome, metabolomics, metabolic alterations, liver diseases, metabolites, hepatology, gastroenterology

## Abstract

Significant changes in gut microbial composition are associated with chronic liver disease. Using preclinical models, it has been demonstrated that ethanol/alcohol-induced liver disease is transmissible through fecal microbiota transplantation (FMT). So, the survival rate of people with severe alcoholic hepatitis got better, which suggests that changes in the makeup and function of gut microbiota play a role in metabolic liver disease. The leaky intestinal barrier plays a major role in influencing metabolic-related liver disease development through the gut microbiota. As a result, viable bacteria and microbial products can be transported to the liver, causing inflammation, contributing to hepatocyte death, and causing the fibrotic response. As metabolic-related liver disease starts and gets worse, gut dysbiosis is linked to changes in the immune system, the bile acid composition, and the metabolic function of the microbiota in the gut. Metabolic-related liver disease, as well as its self-perpetuation, will be demonstrated using data from preclinical and human studies. Further, we summarize how untargeted treatment approaches affect the gut microbiota in metabolic-related liver disease, including dietary changes, probiotics, antibiotics, and FMT. It discusses how targeted therapies can improve liver disease in various areas. These approaches may improve metabolic-related liver disease treatment options.

## Introduction

The gut microbiome influences liver function both directly and indirectly. Gut products reach the liver directly through the portal vein, which transports blood from the intestines. The composition and function of gut bacteria influence metabolites approaching the liver by affecting carbohydrate, protein, lipid, and bile acid metabolism (Caporaso et al., 2010; Oliphant and Allen-Vercoe, 2019; Visconti et al., 2019). Through the portal vein, bacteria, viruses, and fungi in the intestine affect immune cells and molecules that travel to the liver. Gut-liver connections are bidirectional (Madatali Abuwani et al., 2021) and the duodenum receives bile from the liver via bile ducts. Through detergent properties, antimicrobial peptide induction, and immune regulation, bile influences bacterial composition and function (Huttenhower et al., 2012; Wang et al., 2021).

Microbiome therapeutics in liver disease may also target metabolic and immune pathways shared between the gut microbiome and the liver (Gupta et al., 2022b). By entering the intestine through the biliary tree, the liver produces primary bile acids that are deconjugated by intestinal bacteria and further transformed (Qu et al., 2024; Wiefels et al., 2024). The composition of the intestinal bile acid pool, which is largely dictated by the microbiota, affects various aspects of intestinal barrier function, including the mucosal layer, immunological modulation, and tight junction protein integrity (Raimondi et al., 2008; Pavlidis et al., 2015). Short-chain fatty acids (SCFAs: acetic acid, propionic acid, and butyric acid) are products of carbohydrate fermentation and are an important energy source for colonic enterocytes (Wu et al., 2024). Both bile acids and SCFAs, which are products of bacterial metabolism, are important in the regulation of intestinal barrier function and therefore affect the substrates arriving in the liver via the portal circulation (Zeng et al., 2024).

Many molecules cross the intestinal barrier and enter the liver. This is one of many factors that allow pathogens to reach the liver, stimulate macrophages, and stimulate macrophages (Acuna and Olive, 2024). The *Klebsiella pneumoniae* and *Lactobacilli play a significant role in liver metabolisms.* Ammonia is a product of intestinal bacteria that reaches the liver, stimulates the pancreas, and can promote pancreatic growth. The bacterial microbiome of *Klebsiella pneumoniae* Lactobacilli plays a significant role in various liver diseases (Li et al., 2021; Meijnikman et al., 2022). Ammonia is a by-product of intestinal bacteria and may occur.

Several molecules have been implicated in liver disease once they have crossed the gut barrier. The lipopolysaccharide endotoxin is one of several pathogen-associated molecular patterns that can reach the liver, activate macrophages, and promote hepatic fibrosis. Human and animal studies have increasingly demonstrated endogenous alcohol production by microbiota (Cope et al., 2000; Zhu et al., 2013; Yuan et al., 2019).

## Metabolomics platforms in various liver diseases

**Figure 1:**
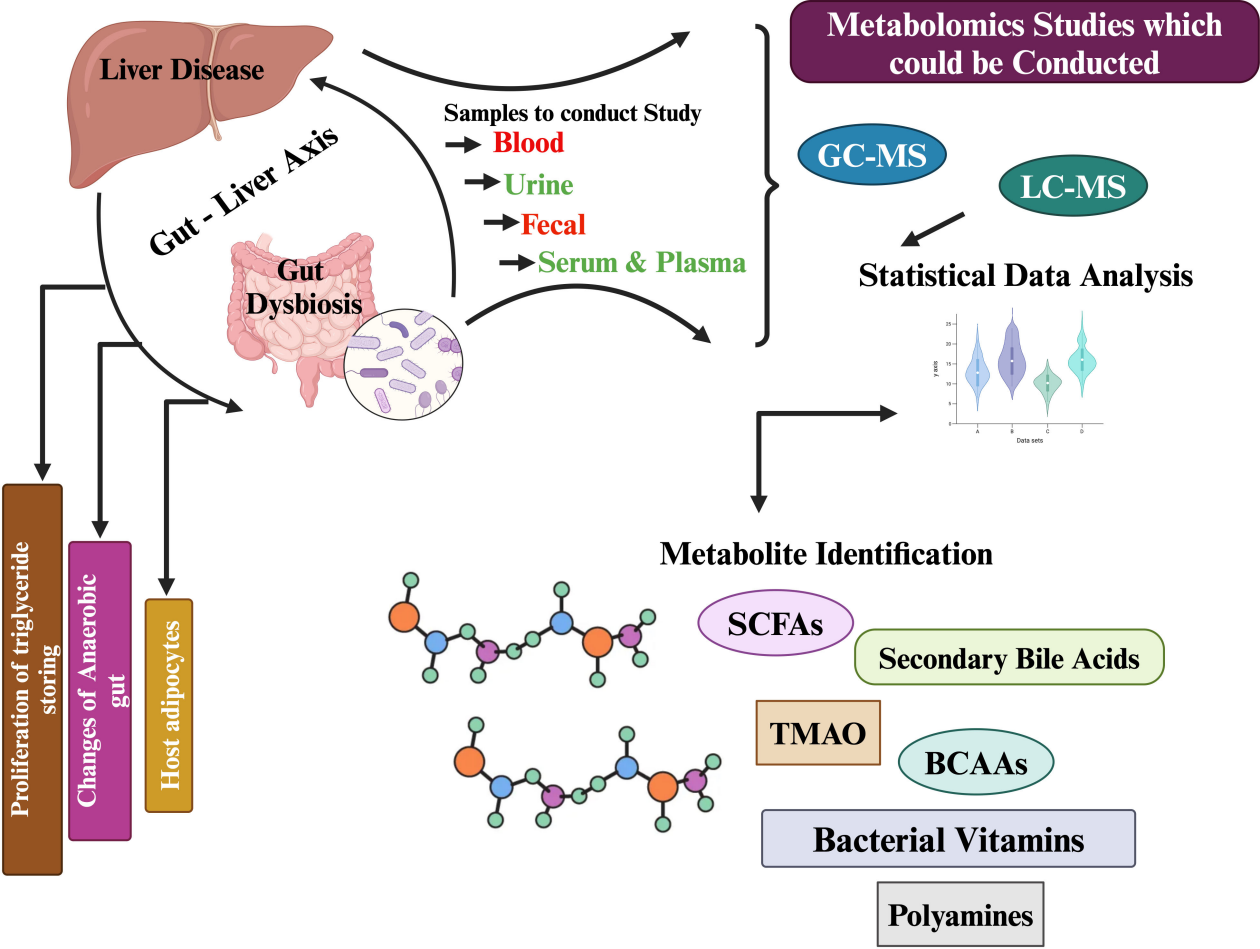
Schematic overview of metabolomics studies in liver diseases.

Biological samples can be analyzed using a variety of analytical platforms, including nuclear magnetic resonance (NMR) and mass spectrometry (MS), the latter coupled to either liquid or gas chromatography (GC-MS). NMR offers high reproducibility and requires less sample preparation than MS techniques, as it is a non-destructive analytical platform (Jaber et al., 2023; Lacalle-Bergeron et al., 2023; Luo et al., 2023; Pekkala, 2023). Using MS, metabolite discrimination and coverage are improved because of its selectivity and sensitivity. It is possible to separate metabolites in a complex matrix before detection, increasing the sensitivity and the sensitivity of detection when combined with a separation technique (Raja et al., 2021). Clinical studies use LC-MS more often than GC-MS because the sample is non-volatile. An individual platform cannot detect, identify, and quantify metabolites completely. Each analytical platform has its own advantages, as well as sensitivity, selectivity, and reliability (Emwas, 2015; Ganesan et al., 2023). A comprehensive metabolite analysis can be achieved by using a combination of NMR and MS, coupled to both liquid and gas chromatography (LC-MS/GC-MS) (Wang et al., 2023; Wang et al., 2023). Combining NMR and MS allows non-destructive analysis of metabolites while also improving the selectivity and sensitivity of analyses of complex matrix metabolites (Zeki Ö et al., 2020; Ganesan et al., 2022). By utilizing both analytical platforms, a more comprehensive understanding of the metabolite profile can be achieved, ensuring more accurate identification, detection, and quantification. Using NMR in metabolite analysis offers high reproducibility and requires less sample preparation, making it a non-destructive analytical platform. On the other hand, MS provides improved metabolite discrimination and coverage due to its selectivity and sensitivity. By combining both NMR and MS, a comprehensive metabolite analysis can be achieved, ensuring more accurate identification, detection, and quantification metabolites in (**Figure 1**) (Madatali Abuwani et al., 2021; Ye et al., 2023; Yu et al., 2023; Yu et al., 2024).

## Gut microbiome and liver diseases

There has been a well-characterized gut microbiome associated with alcohol-related liver disease as well as other chronic liver diseases, showing a reduction in Bacteroides and *Lactobacillus* species and an increase in *Proteobacteria* and *Fusobacteria* (Zhu et al., 2023). A decrease in the *Lachnospiraceae* and *Ruminococcaceae* families was observed in cirrhosis, while significant increases in the *Enterobacteriaceae*, *Alcaligenaceae*, and *Fusobacteriaceae* families are noted. The increase in *Enterobacteriaceae, Alcaligenaceae*, and *Fusobacteriaceae* families in cirrhosis could be due to the disruption of gut microbiota caused by liver damage (Zheng et al., 2023). This disruption can lead to an overgrowth of harmful bacteria from these families, which can contribute to further inflammation and disease progression in cirrhosis. Bacterial microbiota can change with the progression and stage of liver disease, which can lead to further inflammation and disease progression in cirrhosis (Ribeiro et al., 2024). As cirrhosis progresses, harmful bacteria from these families can overgrow. Recent studies have also described fungal dysbiosis. Independent of the stage of liver disease, patients with alcohol use disorder have a decrease in fungal diversity and an increase in Candida species. Identifying the influence of gut microbiota on liver disease progression as well as the influence of progressive liver disease on gut microbiota would be worthwhile (**Table 1**) (Tu et al., 2023; Wang et al., 2024).

**Table 1:** Alterations of gut metabolites in liver diseases.

S. No	Sample type	Species	Pathology	Main findings	Techniques used	References
1.	Blood sample	Human	Acute-on-chronic liver failure (ACLF)	ACLF is strongly linked to an assortment of 38 metabolites, including kynurenic acid, pentose phosphates, and D-glucuronic acid.	Liquid chromatography–mass spectrometry (LC-MS)	Moreau et al., 2020
2.	Fecal sample	Human	Clostridioides difficile infection (CDI)	Bile acid content and leucine digestion resulted in a preliminary metabolomic framework capable of distinguishing clinical CDI from asymptomatic C. difficile colonization.	Gas chromatography–mass spectrometry (GC-MS).	Robinson et al., 2019
3.	Serum	Human	Hepatocellular carcinoma (HCC)	Gut metabolic abnormalities in various liver disorders are linked to pathways for energy metabolism, macromolecular synthesis, and redox balance to protect tumour cells from oxidative stress.	Gas chromatography–time of flight–mass spectrometry (GC-TOFMS)	Gao et al., 2015
4.	Plasma	Human	Liver cirrhotic patients	LTE4 and 12-HHT, both generated from arachidonic acid, created a minimum plasma fingerprint for ACLF.	Liquid chromatography–mass spectrometry (LC-MS)	López-Vicario et al., 2020
5.	Fecal and serum sample	Human	Non-alcoholic fatty liver disease (NAFLD)	The possibility of using gut microbiota for early clinical warning of NAFLD development.	Metagenomics	Leung et al., 2022
6.	Fecal sample	Human	Colorectal Cancer	The fecal metabolic profiles of healthy controls can be differentiated from those of CRC patients, even at an early stage (stage I/II), indicating the potential utility of NMR-based fecal metabolomics fingerprinting as predictors of earlier diagnosis in CRC patients.	Nuclear magnetic resonance (NMR)	Lin et al., 2016
7.	Plasma	Human	Metabolism-associated fatty liver disease (MAFLD)	Choline supplementation can help to alleviate and even prevent hepatic steatosis caused by parenteral feeding.	Computed tomography (CT)	Buchman et al., 1995
8.	Urine	Human	Acute-on-chronic liver failure (ACLF)	Higher Kynurenine Pathway (KP) activity predicted death in patients with ACLF.	Liquid chromatography–mass spectrometry (LC-MS)	Clària et al., 2019
9.	Fecal Sample	Human	Hepatic steatosis	Metabolic profiles indicated favorable relationships for aromatic and branched chain amino acids and glycoprotein acetyls with steatosis and R. Gnavus group, although these metabolites were inversely linked with alpha diversity and Coprococcus.	Metagenomics	Alferink et al., 2021
10.	Fecal sample	Human	Non-alcoholic fatty liver disease (NAFLD)	In NAFLD, the gut microbiome makeup differs, with higher fecal SCFA levels and a greater number of SCFA-producing bacteria. These changes are linked to immunological characteristics of disease development.	High-performance liquid chromatography (HPLC)	Rau et al., 2018
11.	Fecal sample	Human	Crohn’s disease (CD)	Identifying CD metabolites that could serve as diagnostic biomarkers and/or monitoring tools, as well as insight into possible targets for disease therapy and prevention.	High-resolution mass spectra	Jansson et al., 2009

## Prebiotics-based microbial diversity and human health

Health benefits are conferred by prebiotics, which are substrates exclusively used by host microorganisms (Gibson et al., 2017). Hematological stains are a common complication of decompensated cirrhosis, and lactulose is the primary prebiotic for treating it (Bloom and Tapper, 2023). A prebiotic is a substrate used exclusively by host microorganisms that confers health benefits (Gibson et al., 2017). Lactulose is the primary prebiotic for treating HE, a common complication of decompensated cirrhosis (Bloom and Tapper, 2023). In most cases, colonic bacteria ferment lactulose into SCFAs. There are multiple benefits to fermenting lactulose. SCFAs are produced by fermentation of lactulose, which provides nutrients to the intestinal epithelium and reduces gut translocation (Moratalla et al., 2017). A decrease in ammonia production from certain bacteria is attributed to SCFAs, which cause acidification of colonic contents (Sanders et al., 2019). By fermenting lactulose, bacteria can grow more rapidly, pushing other bacteria out of the ecological niche, such as bacteria-producing lipopolysaccharides (Vince and Burridge, 1980; Wang et al., 2019). Ammonia is a substrate used by probiotic taxa as a result of lactulose fermentation (Agostini et al., 1972; Weber, 1979; Vince et al., 1990). Ammonia may translocate across the intestinal epithelium into the colon lumen, be trapped as an ammonium ion, and be expelled in stool due to acidification of colonic contents.

Prebiotics impact gut-derived liver diseases by promoting the growth of beneficial bacteria in the gut. These beneficial bacteria produce metabolites that maintain a healthy gut lining and reduce inflammation, reducing the risk of liver diseases. Additionally, prebiotics stimulate the release of anti-inflammatory molecules and digestive enzymes, which further contribute to preventing and managing liver diseases. Prebiotics stimulate the release of anti-inflammatory molecules such as SCFAs, such as butyrate and acetate. Butyrate in particular has been shown to have potent anti-inflammatory effects, inhibiting the production of pro-inflammatory cytokines and promoting the growth of anti-inflammatory immune cells. These anti-inflammatory molecules help to calm and reduce chronic inflammation, which can be a contributing factor to liver diseases. However, it is important to note that while prebiotics can be beneficial in managing and preventing liver diseases, they are not a magical cure. In some cases, prebiotics may not significantly alter the course of the disease, and individuals may require other interventions or treatments in conjunction with prebiotics. Additionally, prebiotics can interact with other medications or medical conditions, so it is important to consult with a healthcare professional before starting a prebiotic regimen. Prebiotics stimulate the release of anti-inflammatory molecules and digestive enzymes, which further contribute to preventing and managing liver diseases. Specifically, prebiotics promote the growth of beneficial bacteria in the gut, which produce metabolites that maintain a healthy gut lining and reduce inflammation.

Prebiotics are a type of dietary fiber that feeds the beneficial bacteria in the gut. These bacteria play a crucial role in maintaining a balanced gut microbiome and overall health. By promoting a healthy gut microbiome, prebiotics can potentially reduce the risk of gut-derived metabolic liver diseases, such as non-alcoholic fatty liver disease (NAFLD) and cirrhosis (Ince Palamutoglu et al., 2024). Probiotics are defined as preparations or products containing viable, defined microorganisms in sufficient numbers, which alter the microflora of a host compartment by implantation or colonization, and by doing so exert positive health effects on the host (Summer et al., 2024; Teker et al., 2024). Emerging mechanisms probiotics have shown the potential to improve liver function through various mechanisms. Firstly, they can enhance bile acid metabolism, reducing its toxic metabolite levels and protecting the hepatocytes from damage. Secondly, probiotics can stimulate the production of short-chain fatty acids (SCFAs) through gut bacterial fermentation, which has shown hepatoprotective properties (Yoon et al., 2023; Zhang et al., 2023). Lastly, probiotics can modulate the gut microbiota, reducing the growth of harmful bacteria and improving the balance between beneficial and harmful bacteria, ultimately promoting liver health. Probiotics can enhance bile acid metabolism by promoting the growth of specific gut bacteria that can convert bile acids into less toxic forms (Cao et al., 2023). This conversion helps to reduce the risk of liver damage caused by toxic bile acid metabolites. Additionally, probiotics can lower cholesterol levels, which further contributes to improved bile acid metabolism and liver health. SCFAs such as butyric acid, are produced through the fermentation of dietary fiber by gut bacteria. These fatty acids have hepatoprotective properties and can reduce inflammation, oxidative stress, and insulin resistance in the liver. They can also enhance the regeneration of hepatocytes and improve the overall functioning and health of the liver (Zhang et al., 2023; Zhang et al., 2023).

Gut bacteria play a crucial role in the production of SCFAs. When dietary fiber is fermented by gut bacteria, it produces short-chain fatty acids, such as butyric acid. These fatty acids then have a range of beneficial health effects, including protecting the liver from damage and improving its functioning. SCFAs particularly butyric acid, protect the liver through multiple mechanisms. They reduce inflammation by suppressing the cytokines that cause liver inflammation (Ansari et al., 2023; Gao et al., 2023). Additionally, they reduce oxidative stress by neutralizing free radicals and inhibiting lipid peroxidation. They also improve insulin sensitivity, reducing the risk of insulin resistance-related liver diseases. The hepatoprotective properties of SCFAs make them potential candidates for the treatment of liver diseases (Giridhar et al., 2024). They have shown potential in reducing inflammation, oxidative stress, and insulin resistance in the liver, leading to improved hepatocyte regeneration and overall liver health. By targeting these mechanisms, SCFAs may hold promise in the development of novel treatments for liver diseases (Guo and Lv, 2023; He et al., 2023; Hojsak and Kolacek, 2024). Many inflammatory and metabolic disorders have been associated with probiotics.

Some potential applications of probiotics in treating specific medical conditions include:

1. Gastroesophageal Reflux Disease (GERD): Probiotics have been shown to reduce the frequency of GERD symptoms and improve quality of life.
2. Inflammatory Bowel Disease (IBD): Probiotics have been demonstrated to have some beneficial effects on symptoms of Crohn’s disease and ulcerative colitis, the two main forms of IBD.
3. Diarrhea: Probiotics are commonly used to treat and prevent diarrhea caused by a variety of factors, including antibiotics and viral infections.
4. An irritable bowel syndrome (IBS): Probiotics have shown promise in reducing symptoms and improving the quality of life for individuals suffering from IBS.
5. Skin Conditions: Certain probiotics have been used to promote skin health and reduce symptoms of conditions such as eczema and acne.

It is important to note that while there is evidence to suggest the potential benefits of these applications of probiotics, more research is needed to understand their efficacy and long-term outcomes fully.

SCFAs and secondary bile acids are postbiotic products, which are bioactive products of bacteria. In other gastrointestinal conditions, postbiotics have been studied (Golob et al., 2019), but they don’t always produce consistent or uniform positive results (Mortensen and Clausen, 1996; Roda et al., 2007; Abbasi et al., 2022). As of now, there are no clinical trials on SCFAs supplementation in humans. SCFAs supplementation in humans could potentially offer several benefits. It can help improve gut health by promoting a healthy balance of gut bacteria, reducing inflammation, and enhancing intestinal barrier function. Additionally, SCFAs have metabolic benefits, such as reducing insulin resistance, improving weight management, and potentially reducing the risk of chronic diseases like obesity and diabetes (Hsu and Schnabl, 2023). In **Table 2**, we have depicted the therapeutic interventions due to the ingestion of prebiotics in various liver diseases.

**Table 2:** Dietary supplements as therapeutics for liver diseases.

S. No	Disease	Sample	Dietary Intervention	Duration of Therapy	Quantity of Intervention	Findings	Reference
1.	Alcoholic Hepatitis	Fecal sample	Lacticaseibacillus rhamnosus and Lactobacillus helveticus	Seven Days	120 mg/day	The ingestion of L. rhamnosus R0011 and L. helveticus R0052 may restore the gut microbiota in alcoholic hepatitis patients and ameliorate the gut-liver axis.	Gupta et al., 2022a
2.	Alcoholic Hepatitis	Stool culture	Lactobacillus subtilis/Streptococcus faecium	Seven Days	1500 mg/day	Immediate abstinence is the most effective treatment for alcoholic hepatitis. Furthermore, 7 days of oral supplementation with cultured L. subtilis/S. faecium was found to restore gut flora and ameliorate LPS in individuals with alcoholic hepatitis.	Han et al., 2015
3.	Lactose Intolerance & Diarrhoea	Fecal sample	*Bifidobacterium animalis* subsp. *animalis* IM386	Six Weeks		The consumption of the dietary supplements reduced the outcome of diarrhea significantly among the study participants.	Roškar et al., 2017
4.	Lactose Intolerance	Fecal sample	*Lactobacillus acidophilus*	Four Weeks	10 billion (1 X 10^10^) CFU per dose	Lactobacillus acidophilus is safe to eat and improves abdominal symptom scores as compared to placebo in terms of diarrhoea, cramps, and vomiting following an acute lactose challenge.	Pakdaman et al., 2016
5.	Alcohol induced Liver injury	Mouse Model – Fecal sample	Prebiotic - Pectin	Few Week		This dietary supplement improved the alcoholic liver disease by targeting the intestinal microbiota involves the AhR pathway	Wrzosek et al., 2021
6.	Non-alcoholic fatty liver disease	Mouse model – Blood sample	Fructose	12 Weeks	30% fructose	LGG treatment raises hepatic FGF21 expression and serum ADPN concentration, which reduces ChREBP activation via dihydrosphingosine-1-phosphate-mediated PP2A deactivation, and thereby reverses fructose-induced NAFLD.	Zhao et al., 2019
7.	Pouchitis	Human – Fecal sample	Lactobacilli + three strains of bifidobacteria + one strain of *Streptococcus salivarius*.	12 Months	300 billion bacteria/g	The once-daily high-dose probiotic composition is helpful in keeping antibiotic-induced remission in patients with recurrent or refractory pouchitis for at least a year and it is related with a great quality of life.	Mimura et al., 2004
8.	Severe alcohol-associated hepatitis (mAH)		Lactobacillus rhamnosus GG	6 Months	LGG orally once/day	The treatment with LGG resulted in a moderate, but substantial, reduction in MELD at 1 month (primary endpoint) as well as a significant drop in the AST : ALT ratio, a biomarker of AH severity.	Vatsalya et al., 2023
9.	Alcoholic liver disease	Rat model - Blood and Liver tissue	Lactobacillus bulgaricus and Streptococcus thermophilus	6 Weeks	10^8^ cfu/mL	The protective effect against ALD may be attributed to alterations in the gut flora following probiotic-fermented milk consumption.	He et al., 2022
10.	Non-alcoholic fatty liver disease	Human stool samples	*Lactobacillus lactis, Pediococcus pentosaceus*	8 Weeks	10^9^ CFU/g	NAFLD development is linked to metabolic imbalances in SCFAs, bile acid, and indole compounds. These metabolites can be used to precisely identify the disease. L. lactis and P. pentosaceus improve NAFLD progression via influencing gut metagenomics and metabolism, namely the tryptophan pathway of the gut-liver axis.	Yu et al., 2021
11.	Non-alcoholic fatty liver disease	Human fecal sample	*Lactobacillus plantarum + Bifidobacterium bifidum + polysaccharide*	4 weeks	600 mL with distilled water for 3 hours	This study suggests that the LBM combination can be employed as a therapy for alleviating NAFLD by altering the gut microbiota and decreasing insulin resistance.	Wang et al., 2019

## Antibiotics-based microbial diversity and human health

An infant’s gut microbial colonization and resistive profile are influenced by perinatal and peripartum antibiotic use (Zou et al., 2018; Wong et al., 2020). To understand the potential impact of antibiotic administration on offspring during pregnancy, scientists examined the temporal effects of cefoperazone when administered during the peripartum period on the microbiota of both maternal and offspring in an interleukin-10 (IL‐10) ‐ deficient murine model of colitis (Miyoshi et al., 2017).

Cefoperazone-exposed dams had offspring with altered gut microbe communities who were more susceptible to spontaneous and chemically induced colitis (Miyoshi et al., 2017). Similar results were demonstrated by Schulfer et al., who inoculated germ‐free pregnant mice with an antibiotic‐altered microbial community. According to Schulfer et al., 2018 decreased IL-10 proliferates in the offspring after the altered microbial community is transmitted (Schulfer et al., 2018).

There has been some evidence that maternal antibiotic intake during pregnancy alters the composition of the microbial community (Azad et al., 2013; Coker et al., 2020). Fluoroquinolones (norfloxacin and ciprofloxacin), third-generation cephalosporins (G3) (ceftriaxone and cefotaxime), and trimethoprim-sulfamethoxazole (SXT) are recommended for preventing infections in patients with cirrhosis or liver failure. Spontaneous Bacterial Peritonitis (SBP) is a common bacterial infection in patients with cirrhosis. SBP in cirrhosis patients can be caused by the rupture of bacteria-containing ascite pockets or the spread of bacteria from the digestive tract (Puri, 2023; Petruzziello et al., 2024). Risk factors for SBP in cirrhosis patients include advanced cirrhosis, a history of gastrointestinal bleeding, portal hypertension, and the use of gastrointestinal prophylaxis. As well as bacterial overgrowth in small intestines, intestinal permeability increases, and intestinal motility decreases in patients with liver disease. Examples of preventive measures for SBP in patients with cirrhosis include regular monitoring of liver function, proper hygiene and sanitation practices, vaccination against bacterial infections, and antibiotic prophylaxis when necessary (Giridhar et al., 2024). Additionally, maintaining a healthy diet, avoiding alcohol, and managing underlying liver disease can help reduce the risk of SBP. The study conducted by Prado et al. (Prado et al., 2022) also examined whether patients with cirrhosis, who resistant bacteria had earlier colonized, were at greater risk of re-infection by the same strain in the future (Aziz et al., 2022). Increased intestinal permeability, commonly observed in liver disease, allows bacteria to translocate from the intestines to the peritoneal cavity, leading to SBP. The impaired intestinal motility further facilitates the spread of bacteria, making patients more susceptible to infections. Therefore, addressing intestinal permeability and motility is crucial in preventing and managing SBP in patients with cirrhosis. Increased intestinal permeability in cirrhosis patients can lead to bacterial translocation, where the harmful bacteria from the digestive tract enter the peritoneal cavity and cause spontaneous bacterial peritonitis (SBP). This increases the risk of bacterial infections and can lead to serious complications and even death in cirrhosis patients. Additionally, increased intestinal permeability can contribute to developing systemic infections, as harmful bacteria can spread throughout the bloodstream.

Infections caused by spontaneous processes, such as SBP, occur in about 36% of patients with liver cirrhosis (Tay et al., 2021). Third-generation cephalosporins are often used empirically to treat SBP, except in cases where multidrug-resistant organism risk factors apply, where piperacillin/tazobactam is prescribed.

## Translational research in various liver diseases

Specific areas of focus for future translational research in hepatic diseases could include: 1) developing novel therapeutic strategies to target liver diseases, such as gene therapy or targeted drug delivery systems (Rungratanawanich et al., 2023). 2) Understanding the underlying molecular mechanisms of liver disease progression and the development of new diagnostic tools to predict and monitor disease activity. 3) Investigating the role of gut microbiome in liver diseases and its potential impact on the development and severity of liver diseases. 4) Exploring the potential of regenerative medicine approaches, such as stem cell therapy, for the treatment of liver diseases. Examples of liver diseases influenced by the gut microbiome include NAFLD and cirrhosis. Studies have shown that the gut microbiome can contribute to the development of NAFLD, as it can promote the accumulation of fat in the liver (Tanaka and Ui, 2010; Taner et al., 2020). Additionally, the gut microbiome has been implicated in the pathogenesis of cirrhosis, as it can contribute to inflammation and impaired liver function. Examples of gene therapy for liver diseases include the use of viral or non-viral vectors to deliver normal copies of genes that are mutated in liver diseases, or the use of gene therapy to silence the expression of genes involved in liver disease development (Koziel, 2008; Gottlieb et al., 2019). Targeted drug delivery systems, on the other hand, can involve the development of nanoparticles or bioconjugates that specifically target the liver and deliver therapeutic agents directly to the affected cells. Recent advancements in stem cell therapy for liver diseases have shown promising results (Nacif et al., 2018). Researchers have developed techniques to differentiate stem cells into liver cells, which are then transplanted into the liver to replace damaged cells (El-Serag, 2007; Gijbels et al., 2021). This approach has shown potential in the treatment of various liver diseases, including cirrhosis and hepatocellular carcinoma. Additionally, advancements in regenerative medicine have led to the development of bioengineered livers, using a combination of stem cells and biomaterials to create functional liver structures (Ma et al., 2023; Liu et al., 2024; Martini et al., 2024). While further research is needed to fully understand the therapeutic potential of stem cell therapy in liver diseases, these advancements offer hope for potential new treatments. One challenge of targeted drug delivery systems is ensuring the specificity and accuracy of the delivery system. The system must be able to target the right location in the body and avoid delivery to unintended areas, which can be challenging to achieve (El-Serag, 2007; Gijbels et al., 2021). Additionally, the stability and release of the therapeutic agent within the targeted cells can be problematic, as the desired therapeutic effect may be compromised if the drug is released too quickly or too slowly. Furthermore, the systemic delivery of targeted drug delivery systems may be limited by biodistribution and clearance, as the drug may be eliminated or distributed throughout the body before reaching its intended target (Neuman et al., 2015; Huang et al., 2024; Jamshidi et al., 2023).

## Alcohol use disorder and treatment potential of liver diseases

FMT has shown potential in the treatment of alcohol use disorder (AUD). FMT involves the transplantation of a healthy donor’s stool into the recipient’s colon, which can alter the recipient’s gut microbiota and improve overall gut health. This, in turn, could potentially regulate alcohol consumption and withdrawal symptoms, making FMT a valuable tool in the treatment and management of AUD (Neuman et al., 2015). FMT alters gut microbiota by introducing a healthy donor’s stool, which contains a diverse population of microorganisms. These microorganisms establish a new balance in the recipient’s gut, impacting digestion, metabolism, and even brain function (Hwang et al., 2024; Kim et al., 2024). By modifying the gut microbiota, FMT can regulate alcohol consumption and withdrawal symptoms, offering a potential treatment option for AUD (Zafar et al., 2024). Further research should be conducted to examine the effectiveness of FMT at different stages of AUD, such as in the acute withdrawal phase, during ongoing recovery, and in long-term maintenance (McMillan et al., 2024). This would help to determine the optimal timing and frequency of FMT treatments and establish its role in the overall treatment plan for individuals with AUD. Additionally, exploring different delivery methods of FMT, such as capsules or nasal sprays, could further enhance its accessibility and effectiveness in addressing AUD. Further research should be conducted to explore the long-term effects of FMT treatment for AUD (Kim et al., 2024). This would involve tracking the sobriety and overall well-being of individuals who have received FMT treatment for an extended duration, to understand the sustainability of the treatment’s effects and its potential for relapse prevention. Additionally, investigating the effect of FMT on different subgroups within the AUD population, such as individuals with specific genetic traits or co-occurring mental health disorders, would provide more insights into the individualized benefits of FMT (Hu et al., 2023; Hediyal et al., 2024).

## Immunity-based microbiome and metabolome alteration in liver diseases

Liver diseases can disrupt the immune system, leading to a weakened response to infections and an increased risk of infection. Additionally, the immune system can also play a role in the development and progression of liver diseases, as chronic inflammation and immune cells can attack the liver and contribute to liver damage (Won et al., 2021). Specific mechanisms by which liver diseases disrupt the immune system include: 1) Immunodeficiency: Liver diseases can lead to impaired production of immune cells, such as T cells and B cells, leading to a weakened response to infections. 2) Inflammation: Chronic inflammation in liver diseases can recruit immune cells to the liver, which can result in liver damage and impaired immune function. 3) Altered cytokine profile: Liver diseases can cause an imbalance in the production of cytokines, which are signaling molecules that regulate immune responses, leading to dysregulation of the immune system. In addition to the role of chronic inflammation and immune cells in attacking the liver, specific types of immune cells can also directly contribute to liver damage (Schneider et al., 9900). For example, natural killer cells (NKT) and Kupffer, which are both components of the liver’s innate immune system, can release inflammatory mediators and cytokines that can lead to liver damage. Furthermore, activation of T cells and B cells in response to liver antigens can result in autoimmune liver damage and the progression of liver diseases. NKT cells and Kupffer cells play crucial roles in the liver’s innate immune system. While Kupffer cells are responsible for engulfing and removing harmful substances from the liver, NKT cells can produce cytokines that can trigger immune responses (Behary et al., 2021). However, in liver diseases, both cell types can release excessive inflammatory mediators and cytokines, leading to liver damage and promoting the progression of liver diseases. The dysregulation of immune responses in liver diseases can have a profound impact on liver function. The inflammation caused by immune cells and cytokines can lead to liver damage and impaired immune function, making the liver more susceptible to infections. Additionally, the impaired production of immune cells can result in a weakened response to infections, further exacerbating the liver disease. Overall, the dysregulated immune responses contribute to the progression and severity of liver diseases, highlighting the importance of maintaining a balanced immune response for optimal liver health (Ganesan et al., 2022).

The potential consequences of impaired immune cell production include an increased risk of infections, as the body has a weakened ability to fight off pathogens. This can result in more frequent and severe infections, which can further worsen the liver disease (He et al., 2021). Furthermore, the impaired production of immune cells can lead to a compromised immune system, making the individual more susceptible to other types of infections and cancer. Overall, impaired immune cell production can have severe implications for both acute and chronic liver diseases (Wang et al., 2020).

## FMT in liver transplantation recipients: clinical significance

Liver transplantation (LT) is universally acknowledged as the sole therapeutic choice for patients suffering from end-stage liver disease, acute liver failure, and HCC (Ancona et al., 2021). In recent decades, LT has become an established and standard surgical technique for treating liver disorders (Hübscher, 2011). Nevertheless, patients undergoing LT are particularly susceptible to many infections, including *Clostridium difficile infection* (CDI) (Becattini et al., 2016), cytomegalovirus (CMV) infection (Engelmann et al., 2020), fungal infections, and recurrent hepatitis B virus (HBV) infection. A prior cohort analysis indicated that around 19% of deaths that occurred five years after LT were attributed to diverse sources of infection (Annavajhala et al., 2019; Ancona et al., 2021). The primary reason for this is the administration of immunosuppressive medications following liver transplantation, which weakens the immune system’s ability to detect and fight off pathogens. This allows the pathogens to avoid natural immunity and increases the likelihood of infection. In addition, post-LT infection is also linked to pre-transplant infection and other risk factors (Abad et al., 2017). Furthermore, multiple investigations have shown that gut microbiota composition might undergo considerable alterations following LT (Bajaj et al., 2017). Therefore, liver transplant recipients must restore the balance of their gut microbiota by FMT. For example, Schneider et al. documented a case where FMT was performed on a liver transplant patient who had severe *Clostridioides difficile* infection that was further worsened by acute renal injury (Bajaj et al., 2017). Moreover, a meta-analysis of 44 trials (Shogbesan et al., 2018) has provided evidence of the safety of FMT in patients with weakened immune systems. Thus, FMT could serve as a promising treatment approach for treating *Clostridioides difficile* infection following liver transplantation. As far as we know, there has not been a clinical trial that has evaluated the suitability of FMT for treating infectious disorders.

## The future of microbiome and healthy humans

Microbiomes play an important role in the nutrient metabolism and immune regulation in the human body and can directly affect the liver. Lactulose, rifampin, and certain antibiotics are currently being used in liver disease as microbiome-targeted therapeutics. Translational research in many areas is needed before microbiome-targeted therapeutics can be used to treat or prevent liver disease. We should move forward with rigorous randomized clinical trials for microbiome therapeutics such as FMT, consortium products, bacteriophages, and genetically engineered probiotics. These microbiome-targeted therapeutics need further research to better understand their efficacy, mechanism of action, and optimal delivery method.

Patients with HE have completed enrolment in a clinical trial (ClinicalTrials.gov NCT03796598), but results have not yet been released. A patient’s microbiome composition varies even within one liver disease. A prespecified microbiome analysis and stratification, or a *post hoc* analysis of the interaction between therapy and baseline microbiome, will be required for the design of trials and therapy selection to take account of this heterogeneity. One strategy could be to include microbiome analysis as a prespecified endpoint in future trials, allowing for a more comprehensive understanding of the role the microbiome plays in liver disease and response to therapy. Another strategy could be to conduct *post hoc* analyses of the interaction between therapy and baseline microbiome, allowing for a more targeted approach to therapy selection and customization based on individual patient characteristics. Additionally, utilizing standardized protocols for microbiome analysis and ensuring robust sample collection and storage can help minimize heterogeneity and improve the reliability of findings.

## Conclusion

The importance of gut microbiota in host metabolism and immune functions has been summarized in this review, which includes immune development, colonization resistance, and cell signaling. With the help of advanced omics technologies, we are now beginning to understand how the host and microbiota interact complexly. As a result of antibiotics, the bacterial community and the host are disrupted, thereby disrupting the microbial balance. Overall, these approaches offer a promising new set of biomarkers for liver disease diagnosis and therapy. Fecal microbiota transplantation may also have potential as a treatment option for liver diseases. Further research is needed better to understand the safety and efficacy of this approach. Ultimately, these approaches have the potential to revolutionize the way we diagnose and treat liver diseases. As such, they hold great potential for improving the lives of patients with liver diseases.
